# Factors contributing to high-performance competitive swimsuits: a review from materials science and engineering principles

**DOI:** 10.3389/fspor.2026.1902117

**Published:** 2026-10-14

**Authors:** Shengxuan Yuan

**Affiliations:** Beijing 21st Century School, Beijing, China

**Keywords:** biomimetic structures, body compression, competitive swimwear, drag reduction, hydrodynamics, surface modification

## Abstract

High-performance competitive swimsuits are designed and manufactured to enhance athletic performance mainly through the combined effects of drag reduction and optimized body compression. This review synthesizes current research on the key factors in high-performance competitive swimsuits that enhance swimmers' performance, including optimal fiber composition and proportion, warp-knitting techniques, microfiber adoption, surface modification for enhanced hydrophobicity and drag reduction, compression systems, unimpeded thermal exchange, positive sensory and psychological feedback, and advanced manufacturing techniques such as ultrasonic welding and three-dimensional cutting. The analysis indicates that these factors do not operate in isolation but rather function as interdependent and coupled mechanisms, collectively contributing to improved performance, although the size of that contribution has not been quantified. Nonetheless, a small number of factors involve trade-offs that require careful balancing, although their overall influence appears to be relatively minor. This review provides an integrated framework for understanding the complex design principles of high-performance competitive swimsuits and offers insights for future material innovation and engineering optimization.

## 1 Introduction

Swimming has become one of the most widely practiced and competitive aquatic sports worldwide. With its rapid development, swimming has become not merely a recreational activity but also a highly competitive sport that inspires ambition and excitement. Competitive swimmers continuously seek technological and training improvements that enhance speed and hydrodynamic efficiency. This development has encouraged the widespread adoption of high-performance competitive swimsuits among both athletes and the broader public. At major international competitions such as the Olympic Games and the World Championships, high-performance competitive swimsuits are widely used by elite athletes. Nearly all elite competitors wear high-performance competitive swimsuits, such as the Speedo® LZR Racer®, because of the performance benefits they may provide. During the 2008 Beijing Olympics, swimmers wearing Speedo LZR Racer swimsuits, which were made from a mix of woven elastane-nylon and polyurethane and provided artificial buoyancy, were associated with 23 new world records out of 25 and won 94% of the events, according to an industry review (1).

Even after World Aquatics (formerly FINA) banned these non-textile, polyurethane-based suits in 2009, which caused a temporary performance decline worldwide, the newly introduced high-performance competitive swimsuits (such as the Speedo Fastskin® LZR Pure Intent 2.0, and other similar high-performance competitive swimsuits made by other companies) soon restored athletes' performance and have become widely used in recent years. The new high-performance competitive swimsuits no longer provide an advantage through material-driven buoyancy; instead, they primarily offer hydrodynamic benefits through surface engineering and compression structures (2). Although direct independent evidence quantifying the performance enhancement of newer high-performance competitive swimsuits remains limited, Speedo has stated that the LZR Intent 2.0 reduces passive drag and incorporates compression structures, which may improve swimming performance (3). Moreover, the setting of multiple new world records is often taken as evidence that these garments confer a benefit. Although world records depend on many factors—including training, biomechanics, nutrition, and competition environment—the pattern in Table 1 is nonetheless uneven: 34 of the 40 current long-course records have been reset since 2010, whereas six set during the 2008–09 non-textile-suit era still stand. That the sport has moved on in most events, yet not in these six, indicates that the swimwear of that period conferred an advantage large enough to outlast more than 15 years of subsequent advances in training, biomechanics, and nutrition. Those suits are no longer permitted, and no comparable margin should be expected from a textile garment; the question is therefore which factors can still contribute to performance under the current regulations, where the scope for improvement lies primarily in surface engineering and body compression rather than in material buoyancy.

**Table 1:** Current long-course (50 m) world records in swimming, as ratified by World Aquatics on 8 August 2026.

Event	Record	Date set
Men 50 m Freestyle	20.88	20 Mar 2026
Men 100 m Freestyle	46.40	31 Jul 2024
Men 200 m Freestyle^a^	1:42.00	28 Jul 2009
Men 400 m Freestyle	3:39.96	12 Apr 2025
Men 800 m Freestyle^a^	7:32.12	29 Jul 2009
Men 1,500 m Freestyle	14:30.67	4 Aug 2024
Men 50 m Backstroke	23.55	27 Jul 2023
Men 100 m Backstroke	51.60	20 Jun 2022
Men 200 m Backstroke^a^	1:51.92	31 Jul 2009
Men 50 m Breaststroke	25.95	25 Jul 2017
Men 100 m Breaststroke	56.88	21 Jul 2019
Men 200 m Breaststroke	2:05.48	28 Jul 2023
Men 50 m Butterfly	22.27	1 Jul 2018
Men 100 m Butterfly	49.45	31 Jul 2021
Men 200 m Butterfly	1:50.34	21 Jun 2022
Men 200 m Medley	1:52.69	30 Jul 2025
Men 400 m Medley	4:02.50	23 Jul 2023
Men 4 × 100 m Freestyle Relay^a^	3:08.24	11 Aug 2008
Men 4 × 200 m Freestyle Relay^a^	6:58.55	31 Jul 2009
Men 4 × 100 m Medley Relay	3:26.78	1 Aug 2021
Women 50 m Freestyle	23.59	19 Jun 2026
Women 100 m Freestyle	51.71	23 Jul 2017
Women 200 m Freestyle	1:52.23	12 Jun 2024
Women 400 m Freestyle	3:54.18	7 Jun 2025
Women 800 m Freestyle	8:04.12	3 May 2025
Women 1,500 m Freestyle	15:20.48	16 May 2018
Women 50 m Backstroke	26.86	20 Oct 2023
Women 100 m Backstroke	57.13	18 Jun 2024
Women 200 m Backstroke	2:03.14	10 Mar 2023
Women 50 m Breaststroke	29.16	30 Jul 2023
Women 100 m Breaststroke	1:04.13	25 Jul 2017
Women 200 m Breaststroke	2:17.55	21 Apr 2023
Women 50 m Butterfly	24.43	5 Jul 2014
Women 100 m Butterfly	54.33	2 May 2026
Women 200 m Butterfly^a^	2:01.81	21 Oct 2009
Women 200 m Medley	2:05.70	9 Jun 2025
Women 400 m Medley	4:23.65	11 Jun 2025
Women 4 × 100 m Freestyle Relay	3:27.96	23 Jul 2023
Women 4 × 200 m Freestyle Relay	7:37.50	27 Jul 2023
Women 4 × 100 m Medley Relay	3:49.34	3 Aug 2025

Data from World Aquatics (4), retrieved 8 August 2026; only records ratified as of that date are listed. a Record set during the 2008–09 non-textile-suit era and still standing — six of the forty events listed.

Such a major regulatory intervention did not occur again after 2010. Companies were able to design their high-performance competitive swimsuits more flexibly as long as they met the basic requirements set by World Aquatics. This regulatory change encouraged further technological development of Fastskin competitive swimsuits; Speedo also began incorporating technologies adapted from aerospace applications into its high-performance competitive swimsuits, according to the manufacturer's announcement (5).

Researchers have identified several factors that may improve swimmers' performance, including surface engineering, compression structures, and manufacturing techniques. For example, in the latest high-performance competitive swimsuits, compression systems, water-repellent fabrics, and textured fabric zones are among the most common technologies used to enhance performance (2). This conclusion is consistent with how the Speedo Fastskin LZR Pure Intent 2.0 was designed. However, there are also other factors that contribute to performance enhancement. The present review differs from existing ones in scope and in organizing principle. A recent survey of this field covers swimwear as a whole — aesthetics, comfort, speed enhancement, and additional functions such as buoyancy aids and monitoring (2) — whereas the present review is confined to competitive racing suits of the post-2010 regulatory era and examines them using materials science and engineering principles. On that basis the contributing factors are treated as a coupled system in which gains in one property may be traded against losses in another, and are related to the World Aquatics provisions that constrain them. Earlier work on high-speed suits has traced their historical development (6) rather than analyzing them in these terms.

How does materials science relate to this topic? Many conventional materials exhibit limitations in mechanical, chemical, or surface properties that restrict their performance in specialized applications. To improve material performance, both morphological properties and chemical structure may need to be modified (7). Similarly, by modifying the molecular structure of the coating material, the hydrophobicity and other properties of a material can be improved, which may be used in high-performance competitive swimsuits to enhance their properties. For example, Asadollahi et al. (8) describe a practical and economical process to synthesize stable super-hydrophobic surfaces by using an atmospheric-pressure plasma jet technique to develop an organosilicon-based coating.

This study employs a narrative review approach, synthesizing mainly peer-reviewed literature and official regulatory documents to identify the determinants that contribute to high-performance competitive swimsuits. Relevant literature was identified through searches conducted in academic databases such as Google Scholar and peer-reviewed journal repositories. Full texts were obtained from publisher databases such as ScienceDirect and PubMed where available, and search terms combined the review topic with the specific technologies examined, including “competitive swimwear/swimsuit,” “drag reduction,” “compression garment,” “hydrophobicity/hydrophobic coating,” and “biomimetic structure/surface.” Moreover, research that evaluates Fastskin LZR before 2010 would mostly be excluded because World Aquatics had already changed its regulations, but no temporal constraints were applied to research on fundamental scientific principles. For source selection, peer-reviewed journal articles and official World Aquatics documents were treated as the primary evidence base, whereas manufacturer, industry, and trade sources were used only where peer-reviewed evidence was unavailable, principally for product-specific information—such as fiber compositions and proprietary technologies—not reported in the academic literature. Of the 48 sources cited, 26 are peer-reviewed journal articles, 8 are scholarly books or book chapters, 2 are official World Aquatics documents, and 12 are manufacturer, industry, or trade sources; peer-reviewed findings were given the greatest evidential weight, and claims drawn from manufacturer or industry sources are identified as such in the text.

This integrative narrative review examines, from a materials science and engineering perspective, the factors that contribute to high-performance competitive swimsuits and synthesizes these advances within current World Aquatics regulations in the Discussion and Conclusion to provide a reference for manufacturers.

Before examining these factors, it is helpful to define two concepts that recur throughout this review: swimming performance and drag. In competitive swimming, performance is reflected in the swimming velocity (v), since race outcomes are largely decided by speed. Within a biomechanical analysis of competitive swimming (9), this velocity is related to the swimmer's power output (P_o_), propelling efficiency (e_p_), and a drag constant (A):

$$v=\sqrt[{3}]{\frac{e_{p}\cdot P_{o}}{A}}$$ (1)

For a given power output and efficiency, velocity increases as drag decreases; reducing drag is therefore a central goal of high-performance competitive swimsuit design.

The active drag force (F) opposing forward motion is taken to increase with the square of the swimming velocity (F = A·v^2^), a simplification under which A is treated as constant; the same analysis (9) divides this drag into three components:

$$F=F_{f}+F_{p}+F_{w}$$ (2)

Friction drag (F_f_) arises from the friction between the wetted body or suit surface and the water. Pressure drag (F_p_), which, in the analysis of Toussaint and Beek (9), dominates at the high Reynolds numbers prevailing in competitive swimming (Re, the ratio of inertial to viscous forces, of 2.2–2.5 × 10⁶), is governed mainly by the body's frontal cross-sectional area. Wave-making drag (F_w_) results from the water surface deforming into a bow wave and grows steeply as speed increases. The same analysis notes that this contribution may become considerable, particularly when approaching hull speed; wave-making resistance increases with the Froude number, F_r_ = v/√(gL), where v is swimming velocity, L the swimmer's height, and g the acceleration due to gravity (9). The relative contribution of these components therefore varies with swimming speed and with the swimmer's Froude number, and wave-making drag cannot be assumed negligible at racing speeds near the free surface. High-performance competitive swimsuits act chiefly on the first two components: surface engineering of the fabric reduces friction drag, and body compression reduces pressure drag by streamlining the body's form; wave-making drag is governed largely by swimming velocity, body height, and body position, and is therefore little affected by suit materials.

A further distinction should be made explicit before the evidence is reviewed, because the studies drawn on below differ in how directly they bear on racing conditions. As used in the present framework, the force F decomposed in Equation (2) and the drag constant A that appears in Equation (1) both refer to active drag: the resistance encountered while the swimmer is actually swimming (9). Passive drag, by contrast, is measured on a swimmer or a model held in a fixed, non-propelling position and towed through the water at controlled speeds (10, 11); since the propulsive movements of the stroke are by definition absent, such measurements do not reproduce the conditions of active swimming. Part of the evidence below was obtained on fabric specimens rather than on a body, and some of it reports only material properties, such as water contact angle, from which no drag was measured at all. Effects obtained under passive, fabric-level, or material-level conditions cannot be assumed to transfer to active swimming at racing speed, and the basis of each cited effect is indicated below where it bears on how the result should be read.

## 2 Material architecture & composite design

### 2.1 Fiber composition

Many types of human-made fiber, including nylon, polyester, and elastane (also known as spandex; Lycra® is a brand name for elastane), can be used to make high-performance competitive swimsuits. As described in earlier sources (12–14), nylon is a polyamide polymer containing amide groups —(CO–NH)— in its main polymer chain. Polyester fiber refers to fibers in which the long-chain synthetic polymer contains at least 85% ester by total mass. Elastane is a synthetic linear macromolecule composed of no less than 85% polyurethane segments, with alternating hard and soft segments interconnected through urethane bonds –NH–CO–O–.

According to available sources (13, 15), each fiber has its own characteristics. First, nylon has a high tensile strength and better abrasion resistance, and it has good elasticity and superior moisture resistance. It is also soft, lightweight, and allows swimmers to move freely. However, nylon usually has a shorter lifespan because it lacks the ability to resist chlorine or UV radiation. Second, polyester is extremely durable, and it is very long-lasting: it is highly resistant to common pool chemicals such as chlorine, as well as to UV radiation. It also dries quickly, and it can retain its shape well over time. However, polyester-based swimsuits are usually too stiff or less comfortable against the skin for athletes. Third, elastane is lightweight, fast-drying, and allows freedom of movement; it is highly elastic and strengthened, which allows it to stretch up to five times its length and return to the original shape after stretching. All of these properties make elastane a common and suitable material for sportswear.

Because different kinds of fibers have different characteristics, no single fiber is optimal for every requirement; the magnitude of the drag force, for instance, depends on the kind of fiber, as measured on a towed model in water (11). Scientists therefore usually blend several different kinds of fibers to synthesize a new hybrid fiber composite, which can provide better stretchability and improved compression properties (16); reducing passive drag is an additional design objective of such blends.

A high-performance competitive swimsuit can offer different benefits to swimmers depending on its fiber ratios. Therefore, there is no single optimal fiber ratio applicable to all competitive swimmers. The optimal fiber composition depends on athlete-specific factors such as body morphology, stroke technique, and race distance. This dependency reflects the fact that the compression a swimsuit exerts is not uniform but varies with the wearer's body type and swimming posture: the clothing pressure distributed across different body regions has been found in virtual-fitting simulation to change markedly with body shape and swim-related movements (16). Race distance has also been linked to a physiological mechanism, though only as a proposal rather than a finding: a two-page paper that reports no measurements of its own (17) suggests that the strong compression of high-performance competitive swimsuits suppresses peripheral blood circulation, thereby accelerating the anaerobic glycolytic system while suppressing aerobic mitochondrial respiration, an effect that would favor the instantaneous power demands of short-distance events. However, there is a general consensus in the industry regarding the proportions of the constituent fibers, with only minor differences. The studies (16, 18) used two experimental blends, 80% nylon + 20% elastane and 80% polyester + 20% elastane, demonstrating that these two typical fiber ratios benefit athletes by providing better elasticity and durability. Other industry reports reach a similar conclusion. For example, one source (15) suggests that a typical high-quality swimsuit may contain approximately 80% nylon and 20% elastane, a ratio considered to provide a favorable balance of properties. According to this industry source (15), in this blend, strength and a smooth texture are provided by nylon, while stretchability and efficient elastic recovery are attributed to elastane. Therefore, the suit can maintain its shape and close fit over time.

Traditional research suggests that an 80:20 blend of nylon (or polyester) and elastane provides the theoretical best conditions for swimmers to improve their performance, as shown above. However, for elite-level sprinting where maximized compression is the primary goal, leading producers like Speedo, Arena®, and TYR® have pushed the boundary to around 65:35 or 70:30 ratios (nylon/elastane), as shown in Table 2.

**Table 2:** Fabric compositions and manufacturer-specified placements in elite competitive swimsuits.

Model	Fabric (manufacturer designation)	Polyamide/nylon (%)	Elastane (%)^a^	Other (%)	Placement specified by manufacturer	Function stated by manufacturer
Speedo Fastskin LZR Pure Intent	LZR Pure Velocity	65	35	–	Torso (women); waist region (men)	Reproduces a shark-skin surface; hexagonal surface texture reduces resistance in water (19)
Speedo Fastskin LZR Pure Intent	LZR Pure Flex	65	35	–	Underarm/side region (women); waist region (men)	Stretches in all directions, providing flexibility that does not impede movement (19)
Speedo Fastskin LZR Pure Intent	LZR Pure Comprex	75	25	–	Legs (women and men)	Restricts transverse stretch to maintain high compression, while stretching only longitudinally to facilitate leg movement (19)
Speedo Fastskin LZR Pure Valor	LZR Pure Pulse	65	35	–	Single fabric specified; no regional differentiation given in the source	Not stated in the source (19)
Arena Powerskin Carbon Air2	Not separately designated	65	34	Carbon fiber 1^b^	Single-piece exterior shell; three-piece internal lining; horizontal carbon bands	Carbon bands “lock down at a critical stretch point to provide light compression, core stability and power when and where it's needed” (20)
TYR Venzo	Not separately designated	70	30	–	Seamless outer construction (“Seamless Exo Shell”); internal taping (“Endo Max Compression Cage”) over the abdominals, obliques and quadriceps	Prevents water permeating the fabric, giving a higher body position; the compression cage provides support and a snapback effect (21)

Compositions, placements and functions are as stated by the manufacturers in the cited sources. Data for the Speedo models describe the Fastskin LZR Pure Intent and Fastskin LZR Pure Valor as launched in 2019 (19). a Manufacturers use different designations for the same fiber class: elastane (Arena), Lycra spandex (TYR), and polyurethane in Japanese-market documentation, in accordance with local textile-labeling convention. b Arena lists carbon fiber without a percentage; the value shown is the remainder after the stated polyamide and elastane fractions (100−65−34 = 1).

### 2.2 Knitting techniques

It is commonly believed that knitting patterns can affect a fabric's mechanical properties and compression characteristics, which affect swimmers' performance. Therefore, identifying the knitting technique most suitable for high-performance competitive swimsuits is important. The two main knitting techniques are warp knitting and weft knitting. Specifically, warp knitting is a technique in which each warp yarn forms loops oriented along the length of the fabric, with the loops interconnected to form a planar, lengthwise-aligned structure, as described in the book (22); weft knitting is a technique in which a single yarn is used, with loops formed in a widthwise direction, and the fabric is formed by intermeshing the loops, as described in the handbook (23). According to the industrial report (24), warp knitting is the most common and best choice for swimwear fabrics.

Warp knitting provides several structural advantages for high-performance competitive swimsuit fabrics. According to the research (25), composite materials produced using warp knitting show better tensile resistance than composite materials produced using weft knitting. Therefore, warp-knitted swimsuits can form a stronger compression structure for athletes, which provides benefits to athletes according to the research (2). Moreover, industrial reports (24, 26) suggest that warp knitting offers improved durability, stronger structural integrity, exceptional stretch recovery, and high fabric stability compared with weft knitting.

### 2.3 Advanced structural mechanics and composite integration

#### 2.3.1 Microfibers and porosity control

In the production of high-performance competitive swimsuits, manufacturers usually adopt microfibers (known as microdenier or microfilament fibers), which can be made from nylon or other primary materials for producing high-performance competitive swimsuits, rather than the conventional fibers commonly used in everyday clothing. Specifically, microfibers have very small diameters and a fineness typically below 1 denier, as shown in the textbook (27). Such a minute diameter results in a high aspect ratio, which provides higher packing density (27). High packing density reduces textile porosity and therefore lowers water permeability (27). The reduced water permeability helps maintain the hydrophobic behavior of the textile, which leads to drag reduction underwater. Additionally, using microfibers also enhances swimsuit softness (27), potentially improving the swimmer's performance by providing enhanced comfort.

#### 2.3.2 Carbon fiber reinforcement

Carbon fiber can also be used in high-performance competitive swimsuits to improve compression. According to Arena (28), swimsuits made from high-density woven nylon infused with carbon fiber can reduce muscle oscillation and improve body streamlining without sacrificing flexibility or range of motion. This may contribute to enhanced power output, reduced drag through improved posture control, and overall performance gains in competitive swimming. As an example, carbon-wrapped nylon yarn in the form of cages or bends was applied in Arena's Powerskin® series to achieve a higher level of compression compared with common fabrics, while retaining good flexibility and comfort, as shown in the research (2).

#### 2.3.3 Anisotropy with regionalized application

According to the textbook (29), textile materials are highly anisotropic. Anisotropy is an important property for high-performance competitive swimsuits because it allows the fabric to stretch differently in horizontal and vertical directions, providing compression for athletes without restricting their movement, as described in the manufacturer's technical announcement (30). High-performance competitive swimsuits can enhance swimmers' performance by adopting materials that can provide additional compression, such as the carbon fibers discussed above, or a higher proportion of certain fibers, such as elastane, in specific regions of the swimsuit to keep the core muscles firm and supported while allowing free joint rotation or upper-body movement (30). Regional variation in fiber proportions or the use of multiple layers in specific regions also contributes to streamlining body shape, which plays an essential role in reducing turbulence and drag (30). Such regional variation in fiber proportions is reflected in the elite-suit compositions summarized in Table 2.

## 3 Surface engineering

A substantial share of the mechanical work of swimming is expended against hydrodynamic resistance: in one study, competitive swimmers were found to use about 61% of the work per stroke to overcome drag (9). Thus, swimsuits with surfaces that reduce hydrodynamic resistance can play an essential role in improving swimmers' speed and reducing their energy loss during the competition, which has a positive effect on swimmers' performance. The most common and effective method to achieve such an idealized surface is to apply surface engineering, and two of its specific methods are listed below.

### 3.1 Hydrophobic surfaces and coatings

As noted in the research (31), the diffusion and absorption of water into fabrics result in a high degree of hydrodynamic drag on the swimsuit. Nonetheless, an enhanced hydrophobicity of the surface can form a barrier layer that alleviates and slows the process of water diffusion, thereby reducing the fabric-water interaction and freeing swimmers' movement from hydrodynamic resistance. Additionally, highly hydrophobic fabrics may improve swimmers' performance by contributing to buoyancy, stability, and speed. Therefore, enhanced hydrophobicity of the surface of swimsuits may contribute positively to swimmers' performance (31).

The hydrophobicity of swimsuit surfaces can be improved through several material-modification strategies.

As the research (31) shows, a two-step surface modification approach has been developed to enhance the hydrophobicity of polyester (PET) fabrics for high-performance competitive swimsuits. First, the PET fabrics need to be activated. Either chemical saponification using 1 M NaOH at 80 °C for 40 min or an atmospheric air-plasma treatment can be adopted to create reactive carboxyl and hydroxyl groups on the fiber surface. The activated fabrics were then treated with 7% on weight of fabric (owf) amino-silicon (AS) micro-emulsion at pH 5, padded to 80% wet pick-up, dried at 80 °C, and cured at 150 °C for 4 min. This optimal condition resulted in a significantly increased water contact angle and wettability time of up to 80 min—compared to seconds for untreated fabric—along with a dramatic reduction in both wicking distance and percentage. A stable hydrophobic barrier layer was formed on the fabrics through this treatment, without sacrificing or compromising the mechanical or chemical properties of the fabric. Additionally, treated fabrics demonstrate excellent durability after 20 washing cycles, indicating strong reliability. These improvements effectively reduce water absorption and skin friction drag, thus providing added buoyancy, stability, and speed for swimmers (31).

Beyond this laboratory method for improving the hydrophobicity of swimsuits, there are also other commercialized coating techniques. For example, Lamoral® coatings have been applied in Fastskin products. According to the manufacturer's announcement, the latest generation provides water repellency reported to be six times more durable than that of the previous LZR generation (5).

Researchers have developed another method for nylon, the dominant base material in most elite-level high-performance competitive swimsuits. According to the research (32), a three-step chemical modification process has been developed to improve the hydrophobicity of nylon fabric. First, the fabric was pretreated with 1,2,3,4-butanetetracarboxylic acid (BTCA) to increase the number of reactive carboxyl groups on the surface. Tetrabutyltitanate (TBT) was then applied to generate nanoscale Ti-O-Ti particles, which resulted in significantly increased surface roughness. The SEM images reported by Du et al. (32) confirmed this increase; numerous small particles appear on the fabric after modification with BTCA and TBT, revealing an increase in surface roughness, whereas the untreated fabric shows a smooth surface. Finally, octadecylamine (OA) was used to reduce surface energy through its long alkyl chain. The synergistic treatment resulted in a water contact angle of 134°, compared to approximately 90° with OA treatment only. The treated fabric also exhibited strong water repellency (rating 85 out of 100) and hydrostatic pressure resistance (27.64 mbar), with only a slight reduction in tensile strength because of acid hydrolysis. This research strongly demonstrates that with the combination of increased surface roughness and lowered surface energy, hydrophobicity in nylon-based swimwear fabrics can be effectively enhanced.

### 3.2 Biomimetic structure and texture optimization

According to the research (33), after billions of years of evolution, animals have developed outstanding structures to reduce their hydrodynamic drag during underwater motion. Incorporating these structures, such as biomimetic pit structures, into athletes' swimsuits can significantly reduce drag, which enhances swimmers' performance, as the research (34) shows. Two typical biomimetic structures, texture optimization methods, and their main principles are listed below.

The first biomimetic approach is inspired by sharks' dermal denticles. According to the research (35), fine rib-like surface geometries with sharp surface ridges, known as riblets and inspired by sharks, have been developed, which might reduce drag when aligned either parallel or perpendicular to the flow direction. Surfaces with riblet materials—for instance, scalloped riblets, which are similar to the ridges in shark denticles—show an increase of approximately 7% in their self-propelled swimming speed, as the research (35) shows. The study (33) explains that riblets can efficiently delay the occurrence of turbulent vortices, resulting in less momentum transfer and shear stress when they are aligned in the direction of fluid flow. This mechanism may reduce hydrodynamic drag and potentially enhance swimming efficiency.

The second biomimetic structure is inspired by the aquatic-adapted pit structure of the Cybister beetles. According to the research (34), the pit-shaped microstructures that are found on the aquatic beetle Cybister improve hydrodynamic performance by regulating near-wall flow dynamics. These bumpy and uneven morphological characteristics significantly improve hydrodynamic efficiency during swimming via turbulent boundary layer control. In drag measurements on fabric specimens, the hydrophobic small-pit fabric designated as 4# achieved an 84% drag reduction at 400 mm/s (34). By comparison, the current long-course world records correspond to average swimming speeds of about 1.4–2.4 m/s (Table 1), so the measurement was made at well under a third of racing speed. This reduction arises mainly from two effects (34): the Cassie state on the hydrophobic surface, which markedly decreases viscous drag, and the microvortices generated by the pit structures, which delay flow separation and thereby minimize pressure drag. Additionally, the authors also show that matching the pit scale to the thickness of the near-wall viscous sublayer is very important for obtaining optimal drag reduction. Thus, incorporating appropriately sized pit structures can effectively reduce the drag experienced by athletes and therefore enhance swimmers' performance.

## 4 Biomechanical support & compressive mechanics

As studies of land-based exercise show (36, 37), wearing compression sportswear may be beneficial for enhancing overall exercise performance. Specifically, compression sportswear significantly improves speed, endurance, and functional motor performance; wearing compression sportswear might modulate soft tissue movements, such as by restraining muscle oscillations, which can attenuate excessive impact and energy waste; compression garments can exert mechanical pressure on the body surface, thereby compressing, stabilizing, and supporting the underlying tissue. Thus, compression garments are widely used in sports. Compression has also been widely used in the latest high-performance competitive swimsuits for muscle stabilization and body alignment, as it can reduce total passive drag by creating an idealized streamlined shape and a smaller frontal area in the water, as the research (2) shows. To obtain such an ideal compression system, manufacturers either incorporate carbon fibers or vary fiber ratios across different areas, thereby providing additional support to key muscle groups, as the research (2) shows. For example, the manufacturer of the Speedo Fastskin LZR Pure Intent specifies three fabrics of differing composition for different regions of the same garment: a 65:35 nylon–elastane fabric over the torso (women) or waist region (men), and a 75:25 fabric in the leg sections (Table 2) (19).

Researchers have also examined the mechanism by which compression enhances speed. In six elite female swimmers, the suits producing greater compression ranked highest in performance tests, leading the authors to conclude that compression reduces drag, though the differences were not always statistically significant (38). Additionally, as shown in research on land-based exercise (39), compression garments can reduce the feeling of muscle soreness and fatigue when worn during exercise. This may benefit swimmers' performance by reducing subjective fatigue and increasing comfort and confidence.

Compressive swimsuits can offer several potential benefits to swimmers, but experimental data are still required to verify these benefits. It has been proposed that compressive swimsuits may restrict blood flow, reducing aerobic respiration and prompting the glycolytic system to produce ATP more rapidly, and that this shift may enhance explosive power in skeletal muscles and could therefore provide an advantage for swimmers during competitions (17); that proposal, however, is advanced without supporting measurements. Additionally, the research on land-based exercise (37) states that compression sportswear significantly improves speed, endurance, and functional motor performance. Wearing compression sportswear might attenuate excessive impact and energy waste by modulating soft tissue movements, such as by restraining muscle oscillations. These effects are well-proven in land-based sports, whereas they remain scientifically unsubstantiated in swimming or other underwater sports.

## 5 Thermodynamics

According to the research (40), cold exposure degrades athletic performance, reducing strength and power primarily through a decline in muscle temperature, and in swimming it can impair locomotor function through delays in muscle cell membrane repolarization even before general hypothermia is established. According to the research on cold air (41), cooling the skin alone, without any fall in core temperature, shortened cycling time to exhaustion by about 31%, and cooling the core by a further 0.5 °C to 1.0 °C produced an additional reduction of 30% to 40%. This raises the question of whether competitive swimmers need swimsuits with special fabrics or coatings to insulate the body from relatively cold water.

In fact, high-performance competitive swimsuits usually do not contain such insulating fabrics or coatings. According to the official World Aquatics competition regulations (42), the material used must be textile fabric, the maximum total thickness of the material is 0.8 mm, and the material must have a permeability of more than 80 L per m^2 per second, as listed in the section on regulation and compliance below. A garment that is this thin and permeable offers little scope for thermal insulation.

Furthermore, incorporating insulating fabrics or coatings may compromise other properties of the swimsuit, such as hydrophobicity, and adding a new layer of fabric may, to some degree, restrict swimmers' movement by making the swimsuit thicker.

The conditions under which cooling has been shown to impair performance are also far removed from those of competition. According to the research (40), hypothermia is unlikely to be a problem within 30 min of immersion in water even as low as 5 °C, and the deep body temperature of a person in ordinary clothing falls to 35 °C only after about one hour in water at 5 °C and after three to six hours in water at 15 °C. According to the official regulation (42), the water temperature of top swimming competitions must be 25 °C to 28 °C, and even the longest individual pool event is completed in about fifteen minutes (Table 1). The exposure in competition is therefore far warmer and far shorter than the conditions in which cooling impairs performance, and dedicated thermal insulation does not appear necessary under regulated conditions.

These conclusions apply under regulated pool conditions. Hypothermia has been reported in open-water swimmers even at water temperatures of 18 °C to 19 °C and over swims of less than three hours (40), where the thermal problem is different in kind and lies outside the scope of the present review.

## 6 Sensory engineering & psychophysics

According to the research (43), the comfort of sportswear is an important factor affecting one's sports performance, efficiency, and well-being. Specifically, sportswear with poor comfort may negatively affect athletes' performance. Thus, comfortable high-performance competitive swimsuits may also contribute to enhancing athletes' performance during competitions. As the research (43) shows, the four main aspects of sportswear comfort are physiological, ergonomic, skin sensorial, and psychological. The evidence in this section is drawn from research on sport in general and on land-based sportswear rather than on swimming; several of the conclusions applied to competitive swimming therefore remain conjectural.

Physiological comfort mainly refers to a fabric's ability to regulate body temperature, manage moisture, and maintain thermoregulatory balance during exercise, therefore influencing heart rate, metabolic heat, and overall physical performance (43). However, given that thermal insulation does not appear necessary under regulated pool conditions, as the section above shows, physiological comfort is not a central focus in the present discussion of swimsuit-related performance enhancement.

According to the research (43, 44), ergonomic wear comfort is primarily determined by the garment's pattern and fabric elasticity, which affect both fit and freedom of movement. Highly elastic (‘stretch’) garments let athletes move freely and comfortably, and may also reduce the risk of injury or muscle fatigue (39) and lower friction or drag, thereby improving performance (44). These benefits support the use of a high proportion of elastane and of warp knitting, which add elasticity and improve wearer perception. Microfibers can further enhance the compression system and reduce perceived muscle soreness and fatigue during competition.

Skin sensorial comfort refers to the mechanical sensations produced by clothing in direct contact with the skin, such as softness and smoothness, as the research (43) shows. Surfaces with soft and smooth textures may enhance comfort and therefore potentially improve swimmers' performance. Additionally, the psychological aspect encompasses personal preferences, fashion, and ideology, indicating that swimsuits incorporating athletes' preferred patterns or styles may contribute to greater subjective comfort and could potentially influence performance (43). However, further experimental data are still required to validate these two statements.

Furthermore, advertisements or announcements claiming that high-performance competitive swimsuits can lead to faster swimming may make swimmers feel more confident and prepared simply by wearing such a suit, which is known as a placebo effect. A systematic review of 32 studies and 1,513 participants reported a mean effect size of *d* = 0.47 for mechanical ergogenic aids—the category into which a swimsuit falls—and a pooled effect of *d* = 0.38 across all studies, effects that the authors describe as small to moderate (45). The expectation created by such claims may therefore itself make a modest contribution to performance.

## 7 Manufacture & regulatory compliance

### 7.1 Seaming technology

According to the research (6), seaming affects aero/hydrodynamic drag, revealing that the seaming technique used in the production of high-performance competitive swimsuits is an essential factor affecting swimmers' performance. Ultrasonic welding is a physical process that uses mechanical vibrations, usually at a frequency of 20–40 kHz, to soften or melt a thermoplastic material at the joint line, as shown in the research (46). Ultrasonic welding is often used in the production of high-performance competitive swimsuits due to its advantages over other welding processes. These advantages include minimal thermal damage to fibers, the ability to seal without affecting surrounding fabric layers, the creation of an impermeable seam, and the absence of stitch holes in the fabric (46). These advantages help ensure that the seam does not compromise the textile surface, generate additional underwater turbulence, or increase passive hydrodynamic drag that could hinder athletic performance.

An industry source (47) reports that adopting ultrasonic welding helps to reduce drag by up to 6 percent. Thus, ultrasonic welding appears to be one of the most suitable seaming technologies for the production of high-performance competitive swimsuits.

### 7.2 3D cutting

For high-performance competitive swimsuits, a close fit to the swimmer's body is critical for enhancing swimmers' performance; only with precisely fitted swimsuits can benefits such as maintained compression and minimized hydrodynamic drag be effectively achieved. Thus, advanced garment design methods such as 3D body scanning and cutting can improve the fit of the swimsuit to the swimmer's body and thereby maximize functional performance.

According to the research (48), in digital garment engineering, garments can be designed directly on scanned human body models. By defining seam lines on the garment surface and applying surface-flattening algorithms, three-dimensional garment structures can be converted into two-dimensional pattern pieces (48). Experiments show that garments generated through this method fit the human body well (48). This method is also applicable to the production of high-performance competitive swimsuits. A new pattern-making method for swimsuits was developed using three-dimensional (3D) body scanning, as shown in the research (2). The new method demonstrates higher accuracy (2), showing its practical value.

### 7.3 World aquatics regulation and compliance

According to the official World Aquatics competition regulations (40), swimwear used in any Aquatic sport in the Olympic Games or in any World Aquatics Event must comply with certain requirements. Several key requirements that manufacturers should pay particular attention to are listed below:

The Material used for swimsuits must be: “textile fabric(s)” (40).

The maximum total thickness of the Material(s) used is 0.8 mm (40).

Material(s) used must at any point (except on seams, logos or labels) have a permeability value of more than 80 L per m^2^ per second (l/m^2^/s) (40).

At any part of the swimsuit, a maximum of two layers of textile fabric Material is permitted (40).

There are also other important regulations in the official document that manufacturers must consider when designing high-performance competitive swimsuits. However, the four regulations listed above are the ones most closely related to the design and material treatment methods discussed in this paper. In fact, some of the methods presented in this research may be restricted by these regulations, as manufacturers must carefully consider the number and scale of techniques applied in swimsuit production.

## 8 Discussion

Due to official regulations, some techniques and treatments applicable to swimsuit textiles may be restricted, and their full performance potential may therefore not be realized. For example, a multilayer structure in specific regions, such as the torso, can provide greater compression, supporting the core muscles and helping swimmers achieve a more streamlined body shape, which plays an essential role in reducing turbulence and hydrodynamic drag. However, two layers are the maximum number permitted by the official regulations. Thus, this approach to enhancing swimmers' performance in competition is strictly restricted. Additionally, the two biomimetic structures mentioned above may be difficult to reproduce in textile materials. Although researchers found no consistent increase in swimming speed with Speedo fabric (35), Speedo fabric and other surface modification techniques that other dominant high-performance competitive swimsuit brands adopt are probably at the upper limit of surface modification permitted by regulations at the current stage of technological development, suggesting that this type of modification requires compromises and may not fully realize its performance potential now.

Many of the factors mentioned above are closely interconnected and can produce the same effects. First, the adoption of microfibers can reduce porosity, support hydrophobic behavior, and thereby reduce underwater drag, while also enhancing comfort through a softer texture. Second, the compression structure, which may reduce swimmers' fatigue in addition to its other commonly recognized advantages, can be achieved using carbon fiber, warp knitting, multilayer structures (or different fiber ratios), and a high proportion of elastane. Coincidentally, the use of elastane and warp knitting is also associated with enhanced comfort due to the resulting elasticity.

By contrast, very few of the factors impose constraints on one another. For example, microfibers reduce porosity, which limits heat exchange by lowering water permeability. This reduction in heat exchange may slightly restrict swimmers' passive heat dissipation during athletic performance. However, due to the importance of microfibers, the advantages they bring, as well as the fact that thermal regulation plays a relatively less important role in affecting swimmers' performance (as shown below), this negative impact could be negligible. Other potential interactions among factors are not exhaustively discussed here.

While many factors may lead to improvement in swimmers' performance from the perspectives of thermodynamics and psychophysics, the most dominant factors that produce significant enhancement to swimmers' performance may still be those related to drag exerted on swimmers, such as swimmers' body shape and the hydrophobicity of swimsuits. The overall framework integrating these factors and their interrelationships is summarized in Figure 1.

**Figure 1:**
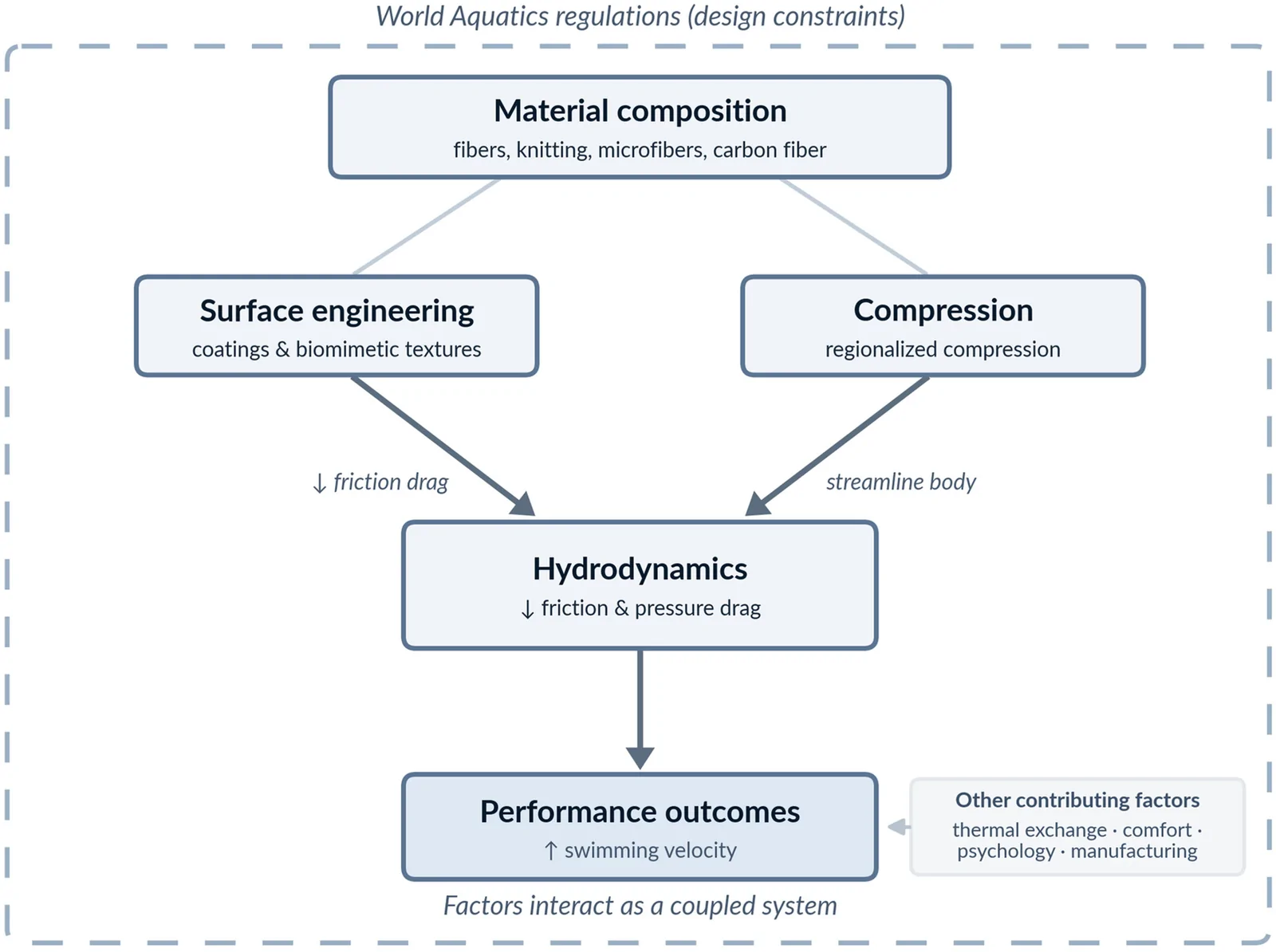
Conceptual framework of the factors contributing to high-performance competitive swimsuit performance. Material composition (fibers, knitting, microfibers, and carbon fiber) provides the fabric basis for two principal design strategies—surface engineering (coatings and biomimetic textures) and body compression—which reduce hydrodynamic drag (chiefly friction and pressure drag) and thereby improve performance. Thermal exchange, comfort, psychological response, and manufacturing contribute secondarily. All design operates within World Aquatics regulations, and the factors interact as a coupled system rather than independently.

### 8.1 Limitations and future directions

While this review provides an overview of high-performance competitive swimsuit technologies, several limitations of the underlying evidence should be acknowledged. First, much of the supporting evidence was obtained under controlled laboratory conditions that differ from actual competition, so the reported effects may not transfer directly to race settings. For instance, the 84% drag reduction reported for pit-shaped biomimetic microstructures (34) was measured at 400 mm/s, well below race speed, and the benefits of hydrophobic surface treatments rest largely on laboratory measures such as water contact angle and a limited number of wash cycles; the durability and real drag reduction of both under prolonged competition use therefore remain to be confirmed. Second, scalability is a practical constraint: translating laboratory-scale surface treatments and biomimetic microstructures into durable, regulation-compliant, mass-produced garments is challenging, and some technologies may require further development before they can be practically adopted. Third, as discussed above, regulatory limits and the absence of a consistent speed benefit for certain surface-modified fabrics further restrict how far these techniques can be realized in practice. Finally, the effectiveness of these techniques in improving performance may differ across swimming strokes owing to differences in water-flow orientation. Overall, further experimental studies under authentic race conditions are needed to confirm the magnitude of these effects.

Future research could then build on such quantified data to arrive at a well-balanced design for high-performance competitive swimsuits, taking account of the trade-offs that exist within the coupled relationships between factors described above. Beyond this, several emerging material and computational directions may further advance high-performance competitive swimsuit design. Nanoparticle and nanostructured coatings could extend the surface-engineering strategies discussed here, potentially offering more durable and finely tunable hydrophobicity and drag reduction. Responsive, “smart” textiles such as shape-memory polymers—which recover a programmed shape in response to stimuli such as body heat—may enable suits that adapt their fit and compression to an individual swimmer's body and movement, thereby improving fit-related streamlining and compression distribution. Computational approaches, including AI-assisted design and digital-twin modeling of the swimmer–suit interaction, could likewise help optimize these interacting factors. As with the technologies reviewed here, however, all such directions will require validation under authentic competition conditions.

## 9 Conclusion

This study shows that the performance of high-performance competitive swimsuits is influenced by multiple interacting factors, including key fiber types and proportions, warp knitting, microfiber use, carbon-fiber reinforcement, regionalized compression structures, surface modification for hydrophobicity and drag reduction, unimpeded thermal exchange, sensory comfort, positive psychological response, advanced manufacturing methods such as ultrasonic welding and 3D cutting, and compliance with World Aquatics regulations. These factors do not operate independently; rather, they interact as a coupled system that collectively enhances swimmer performance. Further research is needed to quantify the effects of specific surface-modification techniques under authentic race conditions, including realistic speeds, motions, and stroke-specific flow environments.
